# Effect of Acute Caffeine Intake on the Fat Oxidation Rate during Exercise: A Systematic Review and Meta-Analysis

**DOI:** 10.3390/nu12123603

**Published:** 2020-11-24

**Authors:** Daniel Collado-Mateo, Ana Myriam Lavín-Pérez, Eugenio Merellano-Navarro, Juan Del Coso

**Affiliations:** 1 Centre for Sport Studies, Rey Juan Carlos University, 28043 Fuenlabrada, Spain; daniel.collado@urjc.es (D.C.-M.); am.lavin.2018@alumnos.urjc.es (A.M.L.-P.); 2 GO fit LAB, Ingesport, 28003 Madrid, Spain; 3 Grupo de Investigación EFISAL, Universidad Autónoma de Chile, Talca 3460000, Chile; emerellanon@uautonoma.cl

**Keywords:** endurance exercise, stimulant, carbohydrate, thermogenic, performance

## Abstract

A number of previous investigations have been designed to determine the effect of acute caffeine intake on the rate of fat oxidation during exercise. However, these investigations have shown contradictory results due to the differences in the exercise protocols used or the co-ingestion of caffeine with other substances. Hence, to date, there is no consensus about the effect of caffeine on fat oxidation during exercise. The purpose of this study was to conduct a systematic review followed by a meta-analysis to establish the effect of acute intake of caffeine (ranging from 2 to 7 mg/kg of body mass) on the rate of fat oxidation during exercise. A total of 19 studies published between 1978 and 2020 were included, all of which employed crossover experimental designs in which the ingestion of caffeine was compared to a placebo. Studies were selected if the exercise intensity was consistent in the caffeine and placebo trials and if these were preceded by a fasting protocol. A subsequent meta-analysis was performed using the random effects model to calculate the standardized mean difference (SMD). The meta-analysis revealed that caffeine significantly (*p* = 0.008) increased the fat oxidation rate (SMD = 0.73; 95% CI = 0.19 to 1.27). This increment was consistent with a significant (*p* = 0.04) reduction of the respiratory exchange ratio (SMD = −0.33; 95% CI = −0.65 to −0.01) and a significant (*p* = 0.049) increase in the oxygen uptake (SMD = 0.23; 95% CI = 0.01 to 0.44). The results also showed that there was a dose–response effect of caffeine on the fat oxidation rate, indicating that more than 3.0 mg/kg is necessary to obtain a statistically significant effect of this stimulant on fat oxidation during exercise. Additionally, the ability of caffeine to enhance fat oxidation during exercise was higher in sedentary or untrained individuals than in trained and recreational athletes. In conclusion, pre-exercise intake of a moderate dose of caffeine may effectively increase fat utilization during aerobic exercise of submaximal intensity performed after a fasting period. However, the fitness level of the participant may modulate the magnitude of the effect of caffeine on fat oxidation during exercise.

## 1. Introduction

Caffeine (1, 3, 7-trymethylxantine) is a methylxanthine alkaloid that is naturally present in the leaves, fruits, and seeds of various plants (coffee, tea, mate, etc.) and is habitually consumed through infusions of these plants. However, the potential benefits of caffeine in terms of enhancing alertness and stamina mean that caffeine is added to numerous dietary supplements. The high presence of caffeine in several foodstuffs, beverages, and supplements has turned caffeine into the most widely used psychostimulant in the world [1]. In sport and exercise settings, caffeine is also widely consumed, with data revealing that 76% of the urine samples of elite athletes obtained after sports competition contained measurable concentrations of caffeine [2]. The effect of caffeine to enhance endurance exercise performance was proposed several decades ago [3]. However, in recent years, the ergogenic properties of the acute intake of low-to-moderate doses of caffeine (from 3 to 9 mg of caffeine per kg of body mass; mg/kg) have been confirmed for anaerobic [4] and strength or power exercise activities [5], and to enhance overall sports performance in several team [6] and individual sports [7].

Interestingly, the ergogenic effect of caffeine reported by Costill et al. [3] during prolonged exercise was associated with a caffeine-induced increase in fat utilization and reduced glycogenolysis, enabling higher muscle glycogen availability late in exercise to prolong time to exhaustion. Although early investigations in the 1970’s confirmed the ergogenic effect of acute caffeine due to increased fat utilization [8,9], posterior investigations indicated that caffeine’s erogenicity was unrelated to enhanced fat utilization or glycogen sparing during exercise [10,11]. Although enhanced fat oxidation is not the main mechanism explaining caffeine’s erogenicity in sport, the use of caffeine and caffeine-containing products to increase fat utilization during exercise of submaximal intensity may be beneficial for other areas of exercise science unrelated to performance, such as body weight loss and changes in body composition. To date, there is a consensus that the antagonistic ability of caffeine depends on adenosine A_1_ and A_2A_ receptors as the main mechanism behind caffeine erogenicity during exercise [12,13]. This mechanism, which produces the blockade to the “fatiguing” effects of adenosine on the central nervous system, explains why caffeine is ergogenic in short-term and high-intensity exercise activities, which do not rely on fat or carbohydrate oxidative pathways. Even though it is well accepted that caffeine’s erogenicity is associated with “central” effects, there is controversy regarding the ability of this substance to shift substrate oxidation in the skeletal muscle during exercise.

Several previous investigations have been designed to determine the effect of acute caffeine ingestion on the fat oxidation rate during aerobic exercise. However, these investigations have shown contradictory results due to the differences in the experimental protocols used. Among these investigations, some protocols have been designed to measure the ergogenic and substrate oxidation effects of caffeine during exercise at the same time [9,14,15]. In this regard, the use of exercise protocols with free-chosen exercise intensity are useful in establishing an increase in exercise performance with acute caffeine intake, but they may offset the effect of caffeine on substrate oxidation, as exercise intensity is a main modulator for the fuels used during endurance exercise. Other investigations have attempted to measure the effect of acute caffeine intake on fat oxidation by measuring the isolated effects of caffeine on oxygen uptake (VO_2_) and on the respiratory exchange ratio (RER) during exercise [16,17]. Although a reduction of the RER indicates a higher reliance of fat oxidation for a given amount of VO_2_, the rate of fat oxidation may also be affected by higher caffeine-induced VO_2_ during exercise [18]. Therefore, small changes in VO_2_ and RER may produce a statistically significant change in the fat oxidation rate during exercise, which may have gone unnoticed in some investigations [19,20]. Other investigations have used multi-ingredient products containing caffeine in addition to other potentially active substances [21,22], making the separation of the effect of caffeine on the fat oxidation rate difficult. Lastly, some investigations have used protocols where caffeine was ingested a few hours after a feeding protocol [23,24]. As caffeine increases exogenous carbohydrate oxidation during exercise [25], the combination of caffeine with pre-exercise feeding may preclude the effect of caffeine on fat oxidation during exercise. Hence, to date, these inconsistencies in the literature have made it difficult to reach a consensus about the effects of caffeine on fat oxidation during exercise. The aim of this investigation was to perform a systematic review and meta-analysis to determine the effect of acute intake of a moderate dose of caffeine (from 2 to 7 mg/kg of body mass) in exercise protocols in which exercise wattage was equal in the placebo and caffeine protocols and carried out after a fasting period.

## 2. Materials and Methods

### 2.1. Search Strategy and Selection of Studies

The current systematic review and posterior meta-analysis were carried out following the guidelines of the Preferred Reporting Items for Systematic Review and Meta-Analyses (PRISMA) [26]. The search for published studies was conducted in the databases PubMed (MEDLINE) and Web of Sciences (including Korean Citation Index-Korean Journal Database, MEDLINE, Russian Science Citation Index, and SciELO Citation Index) from 1 to 7 August 2020. In the search, there was no restriction on the date of publication of the studies. Search terms included the words “caffeine” AND “free fatty acids” OR “fat oxidation” OR “lipid oxidation” OR “substrate oxidation” OR “respiratory exchange ratio” AND exercise. Titles and abstracts were carefully read and screened for subsequent full-text review and data extraction. The search for published studies was independently conducted by two authors (D.C.-M. and J.D.C.), while disagreements between these authors were settled through discussion.

**Figure 1:**
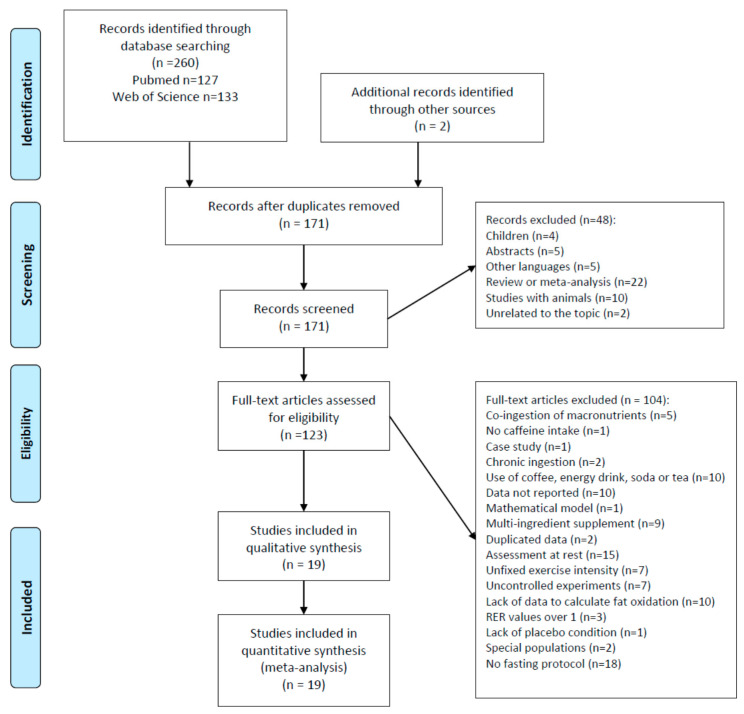
Selection of studies.

To introduce the searched studies in the meta-analysis, the following inclusion criteria had to be fulfilled, with each study involving: (1) testing of the effects of an acute dose of caffeine intake on fat oxidation during continuous or incremental exercise of submaximal intensity, or the effect of acute caffeine intake on VO_2_ and RER during continuous and incremental exercise of submaximal intensity, whereby we selected studies with data on the effects of caffeine on VO_2_ and RER, because fat oxidation can be calculated from these two variables; (2) a crossover design in which an experimental trial with pre-exercise caffeine ingestion was compared to an identical experimental trial with a placebo; (3) information regarding the caffeine supplementation protocol (absolute dose of caffeine in mg or relative dose of caffeine in mg/kg of body mass, timing of caffeine administration, etc.); (4) any source of caffeine was removed from the diet for at least 12 h before the onset of the experiment to wash out dietary caffeine; (5) the exercise protocol was clearly defined in terms of duration and intensity; (6) with exercise protocols in which exercise intensity was identical in all experimental trials; (7) with a fasting period of at least 5 h before the exercise test; (8) written in Spanish or English. The following exclusion criteria were applied to the experimental protocols of the investigation: (1) the use of caffeine doses below 2 mg/kg or equal to and above 9 mg/kg; (2) the absence of a placebo condition; (3) the use of a placebo condition that was not identical to the caffeine trial, except for the use of caffeine (e.g., the use of multi-ingredient complexes, the use of coffee or tea, the use of caffeinated soda); (4) the use of caffeine in combination with fat or carbohydrate; (5) the use of a hot (i.e., >28 °C) or cold (i.e., <5 °C) environment; (6) the use of children as study participants; (7) the use of participants with a previous medical condition or obesity; (8) the use of exercise trials with free-chosen or unfixed exercise intensity. Figure 1 depicts the details of the study selection process and the reasons for the exclusion of studies, which resulted in a total of 19 studies.

### 2.2. Data Extraction

Data extraction was conducted to collect information about participants, intervention, comparisons, outcomes, and study design (PICOS) following PRISMA methodology [26]. Concretely, data on the experimental design, sample size, participant age and level of physical activity, caffeine administration, habitual caffeine intake of the participants, type of exercise, and results of the investigations were gathered independently by two authors (A.M.L.-P. and J.D.C.). In instances where data were presented in a graphical format, images were enlarged to improve the precision of the data estimates and data were extracted using an online application [27]. In those investigations that used a fixed-intensity bout of submaximal intensity, an average of all the fat oxidation rate measurements obtained during the bout was used for analysis. Of note, in experiments that used an exercise protocol with fixed-intensity tests until exhaustion, only measurements obtained at the same time points in the placebo and caffeine trials were used for the analysis. In the investigations with incremental exercise tests, only the data that rendered peak fat oxidation in the caffeine and placebo trials was used for the analysis. In the investigations where fat oxidation rates were not directly presented in the paper, these data were obtained from oxygen uptake and respiratory exchange ratio values by using VCO_2_ using the non-protein respiratory quotient [28].

### 2.3. Quality Assessment of the Experiments

Once the studies were selected for the meta-analysis, the quality of each investigation was evaluated using the Physiotherapy Evidence Database scale (PEDro; [29]). The PEDro scale evaluates the quality of studies using an 11-point scale, with a scoring range from 0 to 10 points, based on the randomization, blinding procedure, statistical analysis, and presentation of the results used in each investigation. The application of the PEDro scale to each study was conducted by A.M.L.-P. and verified by D.C.-M.

### 2.4. Statistical Analyses

An overall analysis was performed including all articles to determine the meta-analytic effects of acute caffeine intake on the fat oxidation rate, VO_2_, and RER during exercise. Moreover, diverse subanalyses of caffeine versus placebo were carried out considering the amount of caffeine administrated in each investigation (≤3.0 mg/kg, 3.1 to 5.9 mg/kg and ≥6.0 mg/kg) and the level of physical activity of the participants involved (trained athletes, recreational exercise practitioners, and sedentary or untrained participants). For the subanalysis of caffeine dosing, we used the intervals mentioned above because they are habitually used to catalogue the magnitude of caffeine intake in terms of exercise performance and side effects [30,31]. The meta-analysis statistics were performed using meta-analysis software (Review Manager Software 5.3, London, UK) with the use of the inverse variance method with random effects and a 95% confidence interval (CI) [32]. The standardized mean difference (SMD) was selected due to the heterogeneity of the exercise tests employed in the selected investigations, which could affect the variability in outcome measurements observed in each study. The level at which an SMD was considered statistically significant was set at *p* < 0.05, while the size of an SMD was interpreted as small with results < 0.4, moderate from 0.4 to 0.7, and large > 0.75 [33]. A standardized measure of homogeneity (I^2^ statistic) was calculated to evaluate the level of heterogeneity in the included sample. I^2^ values between 25% and 50% represented a small amount of inconsistency, I^2^ values between 50% and 75% represented a medium amount of heterogeneity, and I^2^ values > 75% represented a large amount of heterogeneity [34].

## 3. Results

### 3.1. Study Selection

A total of 260 articles were initially obtained in the search and two additional articles were identified by examining the references from relevant articles. After duplicate removal, 171 studies were screened. By reading the title and abstracts, 48 articles were subsequently excluded because they included children as study participants, were not full articles, were not written in English or Spanish, were reviews or meta-analyses, involved animals as study samples, or were completely unrelated to the topic of this review. A total of 123 full-text articles were assessed for eligibility and 104 interventions were excluded for different reasons, including: the co-ingestion of caffeine with fat or carbohydrate; lack of acute caffeine ingestion, a case report; the use of coffee, energy drink, cola, or tea as sources of acute caffeine intake; missing data, the use of a multi-ingredient supplements, duplicated data; assessments not conducted during exercise; unfixed intensity workload; altered experimental conditions; lack of fat oxidation measure; RER values over 1; absence of a placebo condition; the involvement of special populations; and fasting period before the exercise intervention not reported or lower than 5 h, among others. After the selection procedure, 19 studies published between 1978 and 2020 were included in the qualitative and quantitative analysis (Figure 1).

### 3.2. Risk of Bias

The scores for the PEDro scale are reported in Table 1. The mean score for the 19 articles was 8.5 ± 0.9 points, with a range of 6 to 10 points out of a maximal score of 10 points. All of the articles included in the meta-analysis positively fulfilled the statistic criteria (items 10 and 11). Details of the eligibility criteria of participants were only provided by four articles [35,36,37,38] (item 1). The inclusion of only crossover designs in the search strategy implied the administration of caffeine and placebo for the same group or groups of individuals, and in most cases the allocation was performed by randomizing the treatment order (item 2) [3,8,16,17,19,35,36,38,39,40,41,42,43,44,45,46,47,48]. Regarding the blinding process, as assessed by items 5, 6, and 7, most studies used a double-blind design [3,10,16,17,35,37,38,39,40,41,43,44,45,46,47,48], blinding the participants and the therapists. However, only one study included a blinding procedure for the evaluators [40]. In general, the quality of the investigations included in the meta-analysis can be evaluated as high.

### 3.3. Descriptions of the Participants’ Characteristics

The general data for the participants included in this systematic review are depicted in Table 2. The total sample consisted of 203 individuals (147 men, 56 women). In the meta-analysis, only 199 individuals were included due to the lack of data from 4 participants for some of the variables outlined by Clark et al. [44]. In addition, eight participants underwent two different exercise trials at 30 and 50% of VO_2max_ in the study by Engels et al. [35]. Studies involved endurance athletes [3,36,37,39,41,47,50], recreational exercise practitioners [10,17,19,38,40,44,46,49], sedentary individuals [35,37,48], and untrained individuals [16,43,45,50]. The mean age of the study sample was 24.9 years (with averages from 20.5 to 33.0 years). Most of the experiments studied individuals who had low-to-moderate daily caffeine intake (<75 mg/day [40], <100 mg/day [48], 125 mg/day [35], 206 mg/day [38] <220 mg/day [45], <300mg/day [16,36] and <500 mg/day [37]) or employed no regular caffeine users [43]. Still, some studies did not clearly report such information [3,8,10,17,41,47,49].

### 3.4. Descriptions of the Intervention Characteristics

According to Appendix A, 16 out of 19 studies administered caffeine orally based on each participant’s body mass, while an absolute dose was provided for all participants in the remaining three studies [3,39,44]. Overall, the mean caffeine dose administered was 4.56 ± 1.37 mg/kg. In two studies, the caffeine dosage was less than 3 mg/kg [17,44], in three studies it was 3 mg/kg [37,38,40], in nine studies it was between 4.7 and 5 mg/kg [3,8,16,35,36,43,45,46,49], in four studies the dose was 6 mg/kg [10,41,47,48], and in one study the doses were 7 mg/kg [39]. Regarding the administration form, nine investigations used capsules filled with caffeine [10,16,17,39,41,47,48,49] and ten investigations used different caffeine-containing drinks (i.e., lemonade, hot water, decaffeinated coffee, fruit punch with diluted caffeine powder) [3,8,35,36,37,38,43,44,45,46]. All investigations administered the caffeine from 30 to 75 min before the onset of the exercise testing, with most investigations using a 60 min period between intake and testing. Moreover, the participants went through a fasting period from 5 h to 12 h (in ten of the studies [3,8,16,17,35,38,41,43,45,46,48]) or they were asked to fast overnight [10,16,17,35,36,37,38,39,40,41,45,46,48].

As for the exercise testing, 13 studies utilized a cycle ergometer [3,8,10,35,36,37,38,40,44,45,47,48,49], five articles employed a treadmill [16,17,35,39,43], and one used a rowing ergometer [41]. Two investigations used a graded exercise testing until fatigue [44,49], while the remaining studies employed protocols of continuous exercise intensity. In these latter investigations, the exercise intensity was varied, including <10% below lactate threshold [37], 50% the maximal wattage during a previous graded exercise testing ((W_max_), equivalent to 55% VO_2max_) [36], 65% of maximal heart rate (HR_max_) [48], 30% VO_2max_ [46], walking at 30% VO_2max_ and 50% VO_2max_ [35], 55% VO_2max_ [43,45], 60% VO_2max_ [16,38], 60% of VO_2peak_ [40], 64.8% VO_2peak_ [47], 65% to 70% VO_2max_ [8,10], or 80% VO_2max_ [3].

### 3.5. Caffeine Effects during Exercise

**Figure 2:**
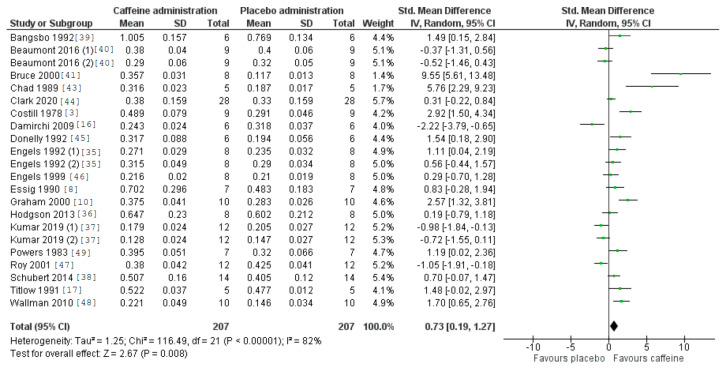
Effect of caffeine ingestion as compared to a placebo on fat oxidation rate during exercise. The forest plot shows standardized mean differences with 95% confidence intervals (CI) for 19 studies that included a measurement of fat oxidation rate or measurements that allowed the calculation of fat oxidation rate (i.e., oxygen uptake and respiratory exchange ratio).

In the meta-analysis, two studies were included by using the original two subgroups of individuals under investigation [37,40], and one study was included where the same sample group conducted two different exercise intensities [35]. Hence, the overall effects of caffeine on the outcomes under investigation were assessed in 22 groups of data from the 19 studies. Figure 2 depicts the effect of acute caffeine intake on the fat oxidation rate during exercise. The effect of caffeine was statistically significant, with a higher fat oxidation rate observed compared to placebo ingestion (*p* = 0.008), with a SMD of 0.73 and 95% CI range of 0.19–1.27.

**Figure 3:**
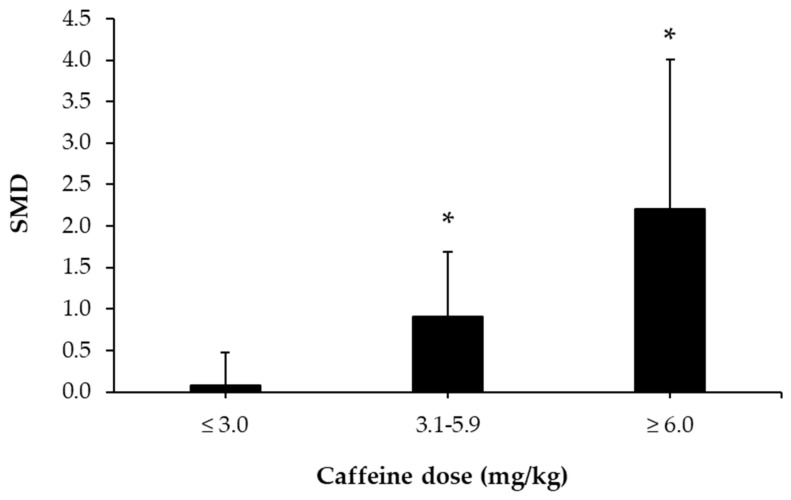
Dose–response effect of caffeine ingestion as compared to a placebo on fat oxidation rate during exercise. SMD values are the standardized mean difference values obtained with a meta-analysis of 19 studies and the bar represents 95% confidence intervals for the SMD for each dosage. (*) The effect of caffeine at this dosage was statistically significant, with a higher fat oxidation rate observed compared to placebo ingestion (*p* < 0.05).

As for the amount of caffeine administrated, the meta-analysis of investigations that administered ≤3.0 mg/kg of caffeine reveal a non-significant effect on fat oxidation rate during exercise compared to placebo (*p* = 0.78). However, in those investigations with doses above 3.0 mg/kg and up to 5.9 mg/kg (*p* = 0.02) or in those with a dose equal to or above 6.0 mg/kg and up to 7.0 mg/kg of caffeine (*p* = 0.03), there was a statistically significant effect of acute caffeine intake on fat oxidation rate during exercise (Figure 3).

**Figure 4:**
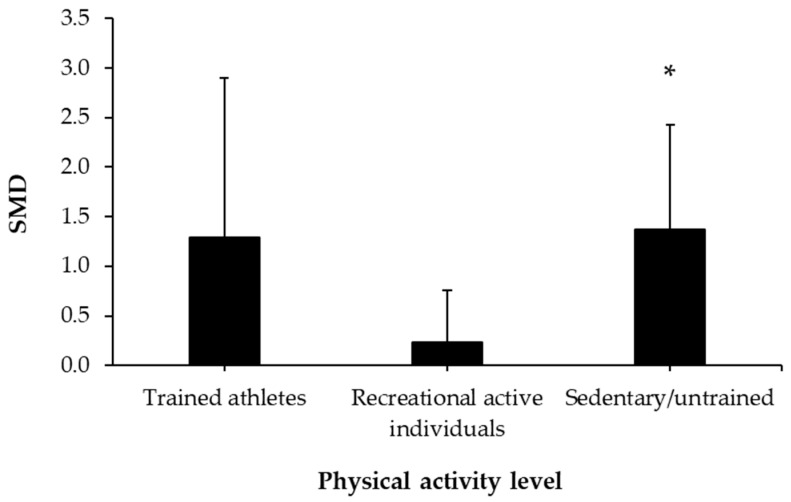
Effect of caffeine ingestion as compared to a placebo on the fat oxidation rate during exercise, depending on the physical activity level of the individuals. SMD values represent the standardized mean differences obtained with a meta-analysis of 19 studies and the bar represents 95% confidence intervals for the SMD in group of participants. (*) The effect of caffeine in this group was statistically significant, with a higher fat oxidation rate observed compared to placebo ingestion (*p* < 0.05).

As for the amount of caffeine administrated, the meta-analysis of investigations that administered ≤3.0 mg/kg of caffeine revealed a non-significant effect on the fat oxidation rate during exercise compared to placebo (*p* = 0.78). However, in those investigations with doses above 3.0 mg/kg and up to 5.9 mg/kg (*p* = 0.02) or in those with a dose equal or above 6.0 mg/kg and up to 7.0 mg/kg of caffeine (*p* = 0.03), there was a statistically significant effect of acute caffeine intake on fat oxidation rate during exercise (Figure 3). Regarding the participants’ levels of physical activity, the meta-analysis of studies revealed a significant effect of caffeine on the fat oxidation rate during exercise only in sedentary or untrained participants (*p* = 0.01; Figure 4), while the effect of caffeine was not significant in trained athletes or in recreational athletes (*p* = 0.12 and *p* = 0.39, respectively).

The heterogeneity was large for the overall effect of caffeine on fat oxidation during exercise (I^2^ = 82%), as well as in the subanalyses performed with the dose of > 6.0 mg/kg of caffeine (I^2^ = 92%) and in the samples of trained athletes (I^2^ = 92%) and sedentary or untrained individuals (I^2^ = 82%). On the other hand, medium heterogeneity values were observed for the effect of caffeine on fat oxidation after taking ≤3 mg/kg of caffeine (I^2^ = 67%) and between 3.1 and 5.9 mg/kg of caffeine (I^2^ = 74%), as well as when analyzing the effect of caffeine on fat oxidation with recreationally active individuals (I^2^ = 62%).

**Figure 5:**
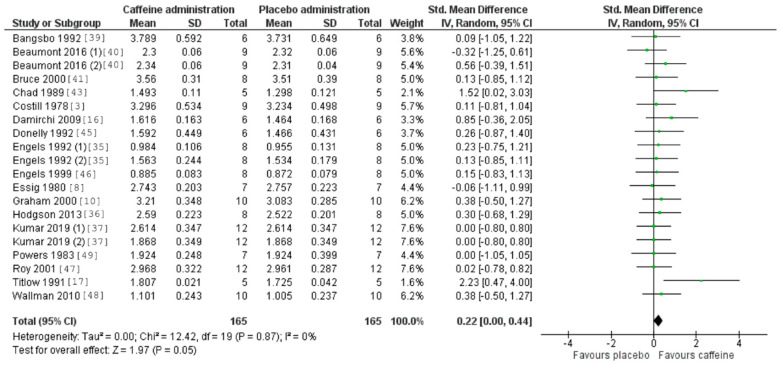
Effect of caffeine ingestion as compared to a placebo on oxygen uptake (VO_2_) during exercise. The forest plot shows standardized mean differences with 95% confidence intervals (CI) for 19 studies that included a measurement of fat oxidation rate or measurements that allowed the calculation of fat oxidation rate (i.e., VO_2_ and respiratory exchange ratio).

**Figure 6:**
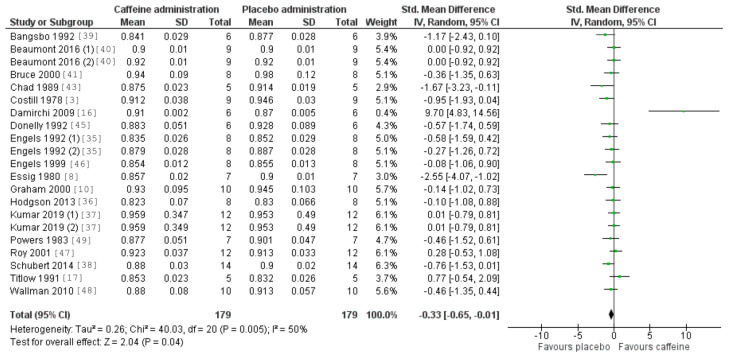
Effect of caffeine ingestion as compared to a placebo on respiratory exchange ratio (RER) during exercise. The forest plot shows standardized mean differences with 95% confidence intervals (CI) for 19 studies that included a measurement of fat oxidation rate or measurements that allowed the calculation of fat oxidation rate (i.e., oxygen uptake and RER).

Figure 5 and Figure 6 also revealed a statistically significant effect of acute caffeine intake on both VO_2_ (*p* < 0.05, with a SMD of 0.22 and 95% CI values ranging from 0.001 to 0.440) and RER (*p* = 0.04, with a SMD of −0.33 and a 95% CI values ranging from −0.65 to −0.01). The heterogeneity was negligible for VO_2_ (I^2^ = 0%) and low–medium for RER (I^2^ = 50%).

## 4. Discussion

The aim of this systematic review and meta-analysis was to summarize evidence on the effects of acute caffeine intake on increasing fat oxidation during exercise of submaximal intensity. The main result of this study indicates that pre-exercise ingestion of caffeine leads to a moderate enhancement of fat oxidation during exercise (Figure 2), as caffeine intake reduced RER (Figure 5) and increased VO_2_ (Figure 6) during exercise protocols with equal exercise wattage in the caffeine and placebo trials. Interestingly, the subanalysis of dosages indicates the need to ingest doses greater than 3.0 mg/kg to obtain such an effect, while there is a dose–response effect on fat oxidation, at least up to doses of 6.0–7.0 mg/kg. Additionally, the fitness level of the participant may alter the magnitude of the effect of caffeine on fat oxidation during exercise, as the magnitude of the caffeine-induced effect of caffeine on fat oxidation was higher in untrained or sedentary individuals. In light of these results, it can be suggested that the acute ingestion of a moderate dose of caffeine may be an effective strategy to increase fat oxidation during submaximal aerobic exercise after a fasting period of at least 5 h.

The effects of acute caffeine intake on shifting substrate oxidation during prolonged exercise has been one of the most discussed effects of this substance. Although it was initially verified by several investigations [3,8,9], subsequent investigations doubted the true ability of caffeine to enhance fat oxidation at a cost of reduced carbohydrate utilization [10,11], arguing that caffeine’s erogenicity is associated with an alternative mechanism, such as antagonism of adenosine receptors [51]. In fact, higher circulating levels of free fatty acids and glycerol [23,45] and lower levels of muscle glycogenolysis [9,52] with acute caffeine intake have been repeatedly reported in the literature, but the efficacy of these physiological changes to explain caffeine’s erogenicity in anaerobic-based and strength exercise is limited. Therefore, it seems clear that the potential effect of caffeine on shifting substrate oxidation is not the main mechanism behinds caffeine’s erogenicity during exercise. Nevertheless, the potential capacity of caffeine to increase fat oxidation during exercise of submaximal exercise intensity may be associated with other benefits beyond exercise and sport performance, such as a more effective body fat reduction in individuals involved in exercise programs aimed at promoting weight loss [53].

The current systematic review used several inclusion criteria to avoid potential confounding variables in the effect of acute caffeine intake on the fat oxidation rate during submaximal exercise. In this regard, we only selected crossover experiments in which the workload used was identical in the placebo and caffeine conditions in order to isolate the effect of caffeine on the substrate oxidation effect of caffeine during exercise from its ergogenic effect. Furthermore, we excluded investigations that used caffeine-containing multi-ingredient supplements, as these supplements contain potentially active substances that may affect the ability of caffeine to shift substrate oxidation during exercise [54,55]. Finally, we set a fasting period of 5 h before exercise to assure that the intake of fat or carbohydrates did not affect caffeine’s effect on substrate oxidation [24,25]. With this search strategy, the systematic review included 19 investigations, the meta-analytical data confirmed the ability of acute caffeine intake to enhance fat oxidation during exercise. Importantly, in 13 out of 19 investigations we calculated fat oxidation rate, as these investigations presented RER and VO_2_ values but they did not actually calculate fat oxidation during exercise. This is important as the meta-analytical approach of this investigation indicates that the effect of caffeine on fat oxidation during exercise is associated with smaller effects in terms of reducing RER and increasing VO_2_. The above-mentioned inclusion conditions for the meta-analysis have likely contributed to improving the identification of the caffeine-induced effects on RER and VO_2_, as previous meta-analysis that did not use these criteria failed to obtain statistically significant differences in these variables between caffeine and placebo [56]. As a practical note for future investigations, we recommend that the study of the ability of caffeine to enhance fat oxidation during exercise should be performed in double-blind and crossover experiments using pure sources of caffeine after a fasting period and by using exercise protocols with identical absolute workloads in the placebo and caffeine trials.

The studies included in this meta-analysis used protocols for caffeine administration, with caffeine intake occurring between 30 and 75 min before the beginning of the exercise protocol. After oral ingestion, caffeine is rapidly and completely absorbed from the gastrointestinal tract, reaching peak plasma concentrations approximately 30–60 min after intake [57,58]. Therefore, measurements of the effect of acute caffeine intake during exercise in the investigations included in this meta-analysis were likely carried out when caffeine concentrations were near peak concentrations in plasma. All investigations included in this meta-analysis consisted of the comparison of one trial with acute caffeine intake before exercise to an experimental trial with the ingestion of a placebo; used pre-experimental standardizations, such as the encouragement of avoiding caffeinated products several hours before the trials; and normalized the time of the day for the experimental trials. Additionally, the exercise intensity used in both trials (i.e., caffeine and placebo trials) was identical, which was controlled by using physiological variables such as VO_2max_, HR_max_, or W_max_ between trials. These controls, which were used in all of the research protocols, suggest that the higher fat oxidation rate found in this meta-analysis is primarily attributed to the acute ingestion of caffeine during exercise of submaximal intensity. However, due to the differences in the types of exercise used (walking, running, rowing, cycling) and the differences in the exercise intensity used to test the ability of caffeine to enhance fat oxidation among experiments (see Appendix A), we were unable to perform an unbiased subanalysis to determine whether exercise intensity modifies the magnitude of the effect of caffeine on the fat oxidation rate. Future investigations should aim to solve this question, as exercise intensity is a main modulator of fat oxidation during exercise. Lastly, the doses of caffeine administered in the experiments ranged from ~2 to ~7 mg/kg. A subanalysis of the effect of caffeine on fat oxidation rate depending on the dose administrated before exercise reflects that there is a dose–response effect on the ability of acute caffeine intake to produce significant effects on fat oxidation during exercise. This subanalysis shows that doses equal or below 3.0 mg/kg do not effectively enhance fat oxidation during exercise, while doses in the range of 3.1 and 7.0 mg/kg are necessary to obtain such effects. This finding may be associated with the need to ingest such doses of caffeine to obtain a significant increment in the plasma epinephrine concentration with respect to a placebo [23]. Nevertheless, further information is required to determine the ideal dosage of caffeine, as the effect of caffeine on substrate oxidation during exercise should be measured in terms of potential side effects produced by caffeine intake [59], particularly at high doses [60].

Caffeine was administered to participants with a wide range of daily caffeine intakes (see Table 2 for further details). Participants could be categorized as low-to-moderate caffeine consumers in most cases [61], but there was a certain heterogeneity regarding habitual caffeine intake among the characteristics of the participants involved in the selected investigations. Recently, it was found that there is a progressive tolerance to the ergogenic [62] and side effects of caffeine [63] when this substance is ingested for 20 consecutive days. Therefore, it remains possible that the differences in habitual caffeine intake among investigations affected the outcomes of this investigation. Additionally, some individuals seeking body fat and weight reductions may use caffeine on a daily basis. In this regard, the efficacy of caffeine ingestion in enhancing fat oxidation may be reduced in individuals who consume moderate-to-high doses of caffeine daily. Therefore, although the current meta-analysis suggests that acute caffeine intake enhances fat oxidation during exercise of submaximal intensity in individuals with low-to-moderate habituation to caffeine, it is still possible that the effects of caffeine in terms of increasing fat oxidation are diminished with chronic ingestion. Future investigations are needed to determine if tolerance to the substrate oxidation effect of caffeine is possibly.

In the current investigation, the ability of caffeine to enhance fat oxidation during exercise reached statistical significance only in those investigations using untrained or sedentary individuals as study participants (Figure 4). In contrast, the magnitude of the effect of caffeine on fat oxidation during exercise was lower and non-significant in statistical terms in individuals with a previous training background (either trained athletes or recreational active individuals). The reasons for the lower effect of caffeine in trained and recreationally active individuals are not evident from our data. In fact, there are previous studies indicating that the ergogenic effect of acute caffeine intake may be higher in trained individuals in comparison with untrained [64] and active individuals [65], although this is not always the case [66]. However, a previous investigation indicated that peak caffeine concentrations following caffeine ingestion (6 mg/kg) tend to be lower in trained compared to active individuals [67], which may be associated with the findings of this investigation. Future investigations should confirm that the ability of caffeine to enhance fat oxidation during prolonged exercise of submaximal intensity depends on the participants’ fitness level, as the lack of statistical significance in groups of trained athletes may be associated with the lower statistical power of the performed subanalysis, depending on the participants’ fitness level.

The current systematic review and meta-analysis presents some limitations that should be discussed in order to increase the applicability of the results. First, several investigations in the field of exercise performance have shown that most but not all individuals experience beneficial responses after acute caffeine intake [7,68,69]. Although factors such as training status and tolerance to caffeine have been suggested as explanations for the existence of “non-responders” to caffeine, the most plausible explanation is the existence of one or several genetic polymorphisms that modify the response to caffeine [70]. In this regard, the most commonly studied polymorphisms are −163C > A present in the *CYP1A2* gene and 1976T > C in the *ADORA2A* gene, however there is a lack of evidence available to clearly argue that either of these two polymorphisms is responsible for the lack of response to caffeine [71,72]. In any case, while acute caffeine intake increases the fat oxidation rate in most individuals, the use of caffeine may not be effective for everyone. The inter-individual variability in response to acute caffeine ingestion suggests that caffeine should be recommended in an individualized manner and continued in those individuals who present benefits with a low prevalence of side effects. Second, the studies included in the meta-analysis used samples of men, women, or mixed samples with individuals of both sexes. Due to the nature of the participants’ characteristics, it was unfeasible to perform a subanalysis by sex. While recent investigations suggest that the response to caffeine during endurance exercise is of a similar magnitude in men and women [64,65], future investigations are needed to ascertain if the ability of caffeine to enhance fat oxidation during exercise is similar in men and women. Third, the I^2^ statistic was superior to 75% for the caffeine-placebo comparison of fat oxidation rates during exercise, indicating a large amount of heterogeneity in the studies included here.

## 5. Conclusions

In summary, the pre-exercise ingestion of a moderate dose of caffeine was found to be effective in increasing the fat oxidation rate during prolonged and submaximal-intensity exercise. Interestingly, there was a dose–response effect of acute caffeine intake on fat utilization during exercise, indicating that a dose higher than 3.0 mg/kg is necessary to obtain a statistically significant effect on the fat oxidation rate during exercise. In addition, the fitness level of the participant may modulate the magnitude of the effect of caffeine on fat oxidation during exercise. In this regard, the magnitude of the effect of caffeine on fat oxidation during exercise was higher in untrained individuals than in individuals with a previous exercise training background (i.e., trained and recreational athletes). The high quality of the selected investigations, the good standardizations employed before the experimental trials, and the avoidance of confounding variables through the selection criteria, such as the use of exercise testing with free-chosen intensity, pre-exercise feeding, and caffeine-containing multi-ingredient products, suggest that this substance may have the ability to enhance fat oxidation during aerobic exercise of submaximal intensity (RER < 1.0) performed after a fasting period.

## Figures and Tables

**Table 1:** Scores for the Physiotherapy Evidence Database (PEDro) scale.

Study/Item	1	2	3	4	5	6	7	8	9	10	11	Total Score
Bangsbo 1992 [39]	N	Y	N	Y	N	N	N	Y	Y	Y	Y	6/10
Beaumont 2016 [40]	N	Y	Y	Y	Y	Y	Y	Y	Y	Y	Y	10/10
Bruce 2000 [41]	N	Y	Y	Y	Y	Y	N	Y	Y	Y	Y	9/10
Chad 1989 [43]	N	Y	Y	Y	Y	Y	N	Y	Y	Y	Y	9/10
Clark 2020 [44]	N	Y	Y	Y	Y	Y	N	Y	N	Y	Y	8/10
Costill 1978 [3]	N	Y	N	Y	Y	N	N	Y	Y	Y	Y	8/10
Damirchi 2009 [16]	N	Y	Y	Y	Y	Y	N	Y	Y	Y	Y	9/10
Donelly 1992 [45]	N	N	Y	Y	Y	Y	N	Y	Y	Y	Y	8/10
Engels 1992 [35]	Y	Y	Y	Y	Y	Y	N	Y	Y	Y	Y	9/10
Engels 1999 [46]	N	Y	Y	Y	Y	Y	N	Y	Y	Y	Y	9/10
Essig 1980 [8]	N	Y	Y	Y	Y	N	N	Y	Y	Y	Y	8/10
Graham 2000 [10]	N	N	Y	Y	Y	Y	N	Y	Y	Y	Y	8/10
Hodgson 2013 [36]	Y	Y	Y	Y	Y	Y	N	Y	Y	Y	Y	9/10
Kumar 2019 [37]	Y	N	Y	Y	Y	Y	N	Y	Y	Y	Y	8/10
Powers 1983 [49]	N	N	Y	Y	Y	N	N	Y	Y	Y	Y	7/10
Roy 2001 [47]	N	Y	Y	Y	Y	Y	N	Y	Y	Y	Y	9/10
Schubert 2014 [38]	Y	Y	Y	Y	Y	Y	N	Y	Y	Y	Y	9/10
Titlow 1991 [17]	N	Y	Y	Y	Y	Y	N	Y	Y	Y	Y	9/10
Wallman 2010 [48]	N	Y	Y	Y	Y	Y	N	Y	Y	Y	Y	9/10

Items: 1: eligibility criteria were specified; 2: subjects were randomly allocated to groups; 3: allocation was concealed; 4: the groups were similar at baseline; 5: there was blinding of all subjects; 6: there was blinding of all therapists; 7: there was blinding of all assessors; 8: measures of at least one key outcome were obtained from more than 85% of the subjects who were initially allocated to groups; 9: intention-to-treat analysis was performed on all subjects who received the treatment; 10: the results of between-group statistical comparisons were reported for at least one key outcome; 11: the study provided both point measures and measures of variability for at least one key outcome. Total score: each satisfied item (except the first) contributes 1 point to the total score, yielding a PEDro scale score that can range from 0 to 10. Y = yes, the item was satisfied in the experimental protocol; N = no, the item was not satisfied in the experimental protocol.

**Table 2:** Summary design and participants.

Study	Design	Age (Years)	Sample Size	Physical Activity Level	Habitual Caffeine Intake
Bangsbo 1992 [39]	RDB	33 (26–39)	6	Long-distance runners with 6 years of experience. Training volume ~58 km/week	6 (0–8) cups of coffee
Beaumont 2016 [40]	RDB	21 ± 2	18 men	Recreationally active practitioners	<75 mg/day
Bruce 2000 [41]	RDB	Not reported	8 men	Well and regularly trained rowers	Not reported
Chad 1989 [43]	RDB	21 ± 1.5	5 women	Untrained (sedentary) individuals	Unhabituated
Clark 2020 [44]	RDB	22.9 ± 0.7 (18–35)	32 men (15) and women (17)	Recreationally active individuals, with less than 150 min/week of exercise in the last 6 months	832 ± 69 mg/week
Costill 1978 [3]	RSB	Men: 22.4; Women: 20.5	9 men (7) and women (2)	Competitive cyclists	Not reported
Damirchi 2009 [16]	RDB	21.8 ± 1.3	6 men	Participants in leisure physical activities (not competitive)	>300 mg/day
Donelly 1992 [45]	RDB	20.5 (SEM 0.5)	6 women	Untrained participants	<220 mg/day
Engels 1992 [35]	RDB	23.5 (21–28)	8 men	Sedentary individuals with VO_2max_ between 30 and 45 mL/kg/min	125 mg/day
Engels 1999 [46]	RDB	26.9 (SEM 1.4)	8 adults (1 woman)	Recreational practitioners (not competitive athletes)	240.9 mg (SEM 57.8) mg/day
Essig 1980 [8]	RSB	24.7 ± 2.2	7 men	Active individuals	Not reported
Graham 2000 [10]	DB	25.7 (20–28)	10 men	Healthy individuals	Not reported
Hodgson 2013 [36]	RSB	41 ± 7	8 men	Cyclists or triathletes who trained 3 or more times per week (90–min/session) in the previous two years	≤300 mg/day
Kumar 2019 [37]	DB	Athletes group: 27.7 ± 5.5; sedentary group: 26.8 ± 7.0	12 athletes and 12 healthy sedentary men (10) and women (2)	Endurance cycling, triathlon, and cross-country athletes practicing >360 min/week of exercise training	<500 mg/day
Powers 1983 [49]	SB	28 (SEM 1.1)	7 men	Recreationally trained bicyclists with 4 to 7 sessions/week.	Not reported
Roy 2001 [47]	RDB	Not reported	12, 7 men and 5 women	Endurance athletes with VO_2max_ > 50 and 60 mL/ki/min for women and men, respectively.	Not reported
Schubert 2014 [38]	RDB	24.9 ± 4.4	14 women (8) and men (6)	Recreational exercise practitioners of ≥30 min of moderate-intensity exercise/day, ≥3 days/week	206 ± 194 mg/day
Titlow 1991 [17]	RDB	25.0 ± 3.6	5 men	Involved in programs of running, cycling, and a variety of sports and games.	Not reported
Wallman 2010 [48]	RDB	22 ± 2	10 women	Healthy sedentary practicing < 20 min of exercise, <3 days/week	<100 mg/day

DB: double-blind; RDB: randomized double-blind; RSB: randomized single-blind; SB: single-blind; SEM: standard error of measurement.

## Appendix A

**Table A1:** Main characteristics of the interventions and protocols of the selected investigations.

Study	Intake	Form	Dose	Protocol	Device	Testing	Fasting
Bangsbo 1992 [39]	30 min before exercise	5 × 100 mg caffeine tablets	500 mg (~7mg/kg)	40 min at 80% VO_2max_	TRE	CONT	Overnight
Beaumont 2016 [40]	60 min before exercise	Capsule	3 mg/kg	60 min at 60% VO_2peak_	CYC	CONT	Overnight
Bruce 2000 [41]	~40–50 min before exercise	Capsule	6 mg/kg	6 min at 80% HR_peak_	ROW	CONT	12 h fast
Chad 1989 [43]	60 min before exercise	Caffeine-containing juice	5 mg/kg	90 min of walking at 55% VO_2max_	TRE	CONT	>5 h
Clark 2020 [44]	~90 min before exercise	Caffeine-containing drink	140 mg (~2 mg/kg)	GXT (M: 35 W/3min and F: 25 W/3 min)	CYC	INC	8 h
Costill 1978 [3]	60 min before exercise	Decaffeinated coffee plus caffeine	330 mg (~4.7 mg/kg)	80% VO_2max_ until exhaustion	CYC	CONT	6–12 h
Damirchi 2009 [16]	60 min before exercise	Capsule	5 mg/kg	30 min at 60% VO_2max_	TRE	CONT	Overnight
Donelly 1992 [45]	60 min before exercise	Caffeine-containing drink	5 mg/kg	90 min at 55% VO_2max_	CYC	CONT	Overnight
Engels 1992 [35]	60 min before exercise	Caffeine-containing drink	5 mg/kg	60 min walking at 30% and at 50% VO_2max_	TRE	CONT	Overnight
Engels 1999 [46]	60 min before exercise	Caffeine-free diet cola plus caffeine	5 mg/kg	60 min at 30% VO_2max_	CYC	CONT	Overnight
Essig 1980 [8]	60 min before exercise	Caffeine-containing drink	5 mg/kg	30 min at ~70% VO_2max_	CYC	CONT	12 h
Graham 2000 [10]	60 min before exercise	Capsule	6 mg/kg	60 min at 70% VO_2max._	CYC	CONT	Overnight
Hodgson 2013 [36]	60 min before exercise	Decaffeinated coffee plus caffeine	5 mg/kg	30 min at 50% W_max_ (~55% VO_2max_).	CYC	CONT	Overnight
Kumar 2019 [37]	40 min before exercise	Caffeine-containing drink	3 mg/kg	25 min at 10% below LT	CYC	CONT	Overnight
Powers 1983 [49]	60 min before exercise	Capsule	5 mg/kg	GXT with 30 W/3 min	CYC	INC	6 h
Roy 2001 [47]	75 min before exercise	Capsule	6 mg/kg	60 min at 65% VO_2peak_	CYC	CONT	10 h
Schubert 2014 [38]	60 min before exercise	Caffeine-containing drink	3 mg/kg	60 min at 60% VO_2max_	CYC	CONT	Overnight
Titlow 1991 [17]	60 min before exercise	Capsules	200 mg (~2.4 mg/kg)	60 min at 60% HR_max_	TRE	CONT	Overnight
Wallman 2010 [48]	60 min before exercise	Capsule	6 mg/kg	15 min at 65% HR_max_	CYC	CONT	12 h fast

CONT: continuous; CYC: cycle ergometer; GXT: graded exercise testing; HR_max_: maximum heart rate; INC: incremental; LT: lactate threshold; ROW: rowing ergometer; TRE: treadmill; VO_2max_: maximum oxygen uptake; VO_2peak_: peak oxygen uptake; W_max_: maximal wattage obtained during a GXT.
